# The inhibitory effect of tongxieyaofang on rats with post infectious irritable bowel syndrome through regulating colonic par-2 receptor

**DOI:** 10.1186/1472-6882-13-246

**Published:** 2013-10-02

**Authors:** Xuguang Hu, Xiaojun Zhang, Bin Han, Weijian Bei

**Affiliations:** 1 School of Traditional Chinese medicine, Guangdong Pharmaceutical University, Guangzhou, 510006, P.R China; 2 School of Pharmacy, Guangzhou University of Chinese Medicine, Guangzhou, P.R China

**Keywords:** Irritable bowel syndrome, Tong-Xie-Yao-Fang, PAR-2 receptor

## Abstract

**Background:**

The aims of this study were to evaluate the effect and mechanism of a traditional Chinese medicine formula: Tongxieyaofang (TXYF) on Rats with Post Infectious Irritable Bowel Syndrome (PI-IBS).

**Methods:**

SD male rats in adult were used to model PI-IBS and treated with TXYF at three dosage for 14 consecutive days, and then visceral sensation and the frequency of stool in PI-IBS rats were investigated. In addition, the contents of SP, TNF- α and IL-6 in colonic mucosal were analyzed by ELISA. Moreover faecal serine protease activity and PAR-2 mRNA expression were measured by ultraviolet spectrophotometry and RT-PCR, respectively.

**Results:**

Our study showed that TXYF attenuated visceral hyperalgesia and inhibited stool frequency in Campylobacter-stimulated Post Infectious Irritable Bowel Syndrome (PI-IBS) rats. Furthermore, TXYF decreased the colonic SP, TNF- α and IL-6 content in PI-IBS rats. In addition, the up-regulated colonic mucosa PAR-2 mRNA expression in PI-IBS rats was significantly suppressed by orally TXYF.

**Conclusions:**

TXYF attenuated PI-IBS symptom by attenuating behavioral hyperalgesia and anti-diarrhea, the underlying mechanism was mediated by inhibiting PAR-2 receptor expression, reducing the levels of SP, TNF- α and IL-6 in colonic mucosa and decreasing faecal serine protease activity.

## Background

Post-infectious irritable bowel syndrome (PI-IBS) was defined as IBS symptoms occur after an initial episode of acute gastrointestinal infection. Actually, studies demonstrated that 15–32% of enteric infections led to the generation of new IBS symptoms [1,2], and mounted evidence indicated that previous GI infection or inflammation played an important role in the pathogenesis of IBS [3,4]. Thus, increased attention has been paid to PI –IBS recently, because it was believed that the clear onset and well-defined pathophysiological changes could help us explain the IBS [5].

Tong-Xie-Yao-Fang (TXYF), a prescription in traditional Chinese medicine (TCM), has been widely used to relieve symptoms associated with IBS [6]. TXYF composed of atractylodes rhizome, white peony root, dried old orange peel and ledebouriella root (LR) [7]. Our preliminary experimental results demonstrated that TXYF had a significant analgesic effect on IBS rats through regulation the 5-HT in the periphery and CRF in the center [8]. The preliminary clinical study showed that TXYF had the anti-pain and anti-diarrhoea effect on the D-IBS patents [9]. Our recently experimental results also showed that TXYF had the hyperalgesia and anti -diarrhoea effect in mice [10]. But the precise mechanism of TXYF has not been fully clear.

Protease activated receptor 2 (PAR-2) is a serine protease receptor which widely distributed in a variety of cells of the digestive tract. Activation of PAR-2 receptor is involved in gut sensation, motility disorders and the intestinal epithelial barrier abnormal. Clinic study showed that there were high level of fecal fluid serine enzyme (a protease activation of PAR-2 receptor) and increased contents of SP, IL-6 and TNF- α in serum of PI-IBS patients. Serine enzyme induced visceral pain sensitivity and intestinal permeability in mouse, and activated the intestinal PAR-2 receptor [11]. Therefore, PAR-2 is suggested to be a new target for drug treatment of IBS [12].

To examine the effect of TXYF and elucidate its mechanism on PI-IBS model, PI-IBS rats were orally treated with TXYF for 14 consecutive days, and then visceral sensation and the frequency of stool were investigated. In addition, the contents of SP, TNF- α and IL-6 in colonic mucosal were analyzed by ELISA. Moreover faecal serine protease activity and PAR-2 mRNA expression were measured by ultraviolet spectrophotometry and RT-PCR, respectively.

## Methods

### Material

Alosetron was purchased from Sigma-Aldrich (St. Louis, MO, USA); SP, TNF- α and IL-6 ELISA measurement kits were obtained from Jiangcheng Bioengineering Institute (Nanjing, China). Universal RT-PCR measurement kit was obtained from Dingguo Bioengineering Institute (Beijing, China), PAR-2 PCR primers was synthesis by Shanghai Sangon Biological Engineering Technology & Services Co., Ltd. (Shanghai, China).

### Animals

Sprague–Dawley rats were obtained from the Guangdong Experimental Animal Center. All the rats were individually housed in cages containing bedding material on a 12:12-h light–dark cycle (lights on at 08:00) and provided with food and water ad libitum. The studies were performed in accordance with the proposals of the Committee for Research and Ethical Issues of the International Association and were approved by the Committee on the Use of Human and Animal Subjects in Teaching and Research, Guangdong Pharmaceutical University.

### Development of PI-IBS model

The PI-IBS rat model was developed according to previous report [13]. 50 Sprague–Dawley rats were gavaged with a 1 mL solution containing C. jejuni 81–176 at 10^8^ CFU/mL in Brucella broth. Following gavage, all the rats were housed at five per cage. In the first 3d after gavage, stool was collected to verify that intestinal colonization in C. jejuni rats had occurred.

After 90% of the C. jejuni rats no longer had detectable C. jejuni in the stool, they were considered to be in the post-infectious time period. At this point, they were housed for three additional months. During this time, the stool was also cultured to determine if there was any lingering case of Campylobacter.

### Campylobacter gavage

The C. jejuni 81–176 strain used in the gavage of the rats was obtained from freezer stocks, plated on selective media, and incubated for 36 h under microaerophilic conditions at 42°C to create a bacterial lawn. This lawn was then harvested from these plates and suspended in Brucella broth. The concentration of bacteria was estimated spectropho- tometrically and confirmed via serial dilution and plating on selective media. In the 30 min prior to Campylobacter gavage, rats were gavaged with a 1 mL solution of 5% sodium bicarbonate using a ball-tipped inoculating needle. This was done to neutralize gastric acid to increase the likelihood of intestinal colonization of the pathogen. Subsequently, a 1mL suspension of C. jejuni in Brucella broth was administered by gavage.

### Preparation of TXYF

TXYF extract was supplied by TianJiang pharmacy company, (Lot No. 1203051, Jiangyin, China). The Compositions of TXYF were as following: 6 g Rhizoma Atractylodis Macrocephalae, 4 g Radix Paeoniae Alba, 3 g Pericarpium Citri Reticulatae and 3 g Radix Sapshnikoviae as shown in Table 1. The raw materials in TXYF all identified by TianJiang pharmacy company. TXYF extract was examined according to the quality control criteria as previous report [14]. 1 g extract of TXYF was equivalent to 5.3 g raw materials in TXYF. The contents of indicator constituents in TXYF extract were as following: paeoniflorin, 38.35 mg/g extract and atractylenolide-1,47.76 mg/g extract. The extract was freshly prepared with distilled water at the desired concentrations just before use. TXYF 4.8, 2.4 g/kg and 1.2 g/kg were defined as high dosage, medium dosage and low dosage, respectively.

**Table 1:** Herbal compositions of TXYF^*^

**Herbal name (Latin name)**	**Quantity (dry, g)**
Rhizoma Atractylodis Macrocephalae	6.0
Radix Paeoniae Alba	4.0
Pericarpium Citri Reticulatae	3.0
Radix Sapshnikoviae	3.0

*Four grams of extract from the prescription are obtained in the manufacturing process, and are clinically used as the daily dose.

### Experimental design

The experiment aimed to test whether TXYF could attenuate visceral hyperalgesia and remedy diarrheal in Campylobacter-induced PI-IBS rats. Rats were divided into 6 group with 10 rats in each group. The Normal rats (Group 1) were treated with distilled water. The PI-IBS rats were randomly divided into 5 groups and treated with distilled water (Group 2), TXYF-H (Group 3), TXYF-M (Group 4), TXYF-L (Group 5) and Alosetron (Group 6) respectively. After 2 weeks treatment, rats were used to measure pain threshold pressure and stool frequency, and the stool was collcectd for total faecal serine protease activity measurmen. Moreover, all the rats subsequently were sacrificed to collect colon samples. A 6 cm long proximal colon (1–2 cm from caecum) was harvested and divided into 2 parts, the proximal was collected for SP, TNF- α and IL-6 ELISA, the transverse colon mucosa was also collected for PAR-2 mRNA assay.

### Examination of abdominal withdrawal reflex and stool frequency

Examination of the abdominal withdrawal reflex (AWR) was measured using the procedure modified from previous reports [15]. Briefly, rats were lightly anesthetized with diethyl ether, and a balloon (4 cm in length, made from the finger of a latex glove) was inserted through the anus into the rectum and descending colon, attached to a Fogarty catheter. The open end of the balloon was secured to the catheter with thread and wrapped with tape (1 cm wide). Prior to use, the balloon was inflated and left overnight so that the latex could stretch and the balloon could become compliant. The balloon was inserted so that the thread was approximately 1 cm proximal to the anal sphincter, and was held in place by taping the tubing to the tail. The catheter was attached via a connector to a sphygmomanometer pump and a pressure gauge. The rats were then housed in small Lucite cubicle (20 × 8 × 8 cm) on an elevated plexiglas platform and allowed to wake up and adapt for 30 min. Colorectal distention (CRD) was produced by rapidly inflating the balloon to the desired pressure (20, 40, 60, or 80 mm Hg) for a duration of 10 s. Stimuli were applied in an ascending graded manner (spaced by 4 min). The AWR, an involuntary motor reflex similar to the visceromotor reflex, was recorded. The AWR score was assigned as follows: 0 = no behavioral response to distension, l = brief head movements followed by abdominal muscle without immobility, 2 = contraction of lifting of abdomen, 3 = lifting of abdomen, 4 = body arching and lifting of pelvic structure. Measurements of the AWR by visual observation were reproduced by two blinded observers. The pain threshold pressure (PTP) is defined as the stimulus pressure that evokes a visually identifiable contraction of the abdominal wall. Increasing pressure was applied in steps of 5 mm Hg lasting 30 s until pain behavior was displayed or until 80 mmHg was reached to avoid lasting damage to the animals. The stool frequency in 24 hours of all animals was measured after the PI-IBS rat model was treated for two weeks.

### Measurement of faecal serine enzymatic activities

To measure total faecal serine protease activity, supernatants of faecal homogenates (5 ml) were incubated with 1 ml of reaction buffer (0.15 M NaCl and 20 mM Tris–HCl, pH 8.3) and 1 ml of 0.5% azocasein at 40°C. The reaction was stopped after 20 min with1 ml of 10% trichloracetic acid. Following centrifugation, absorption of the clear supernatant was measured at 366 nm.

### Measurement of SP, TNF- α and IL-6 in colonic mucosa

Rat colon was washed repeatedly with ice-cold saline solution. The mucosa layer was scraped and homogenized. After centrifuge at 10,000G for 10 min at 4°C, the supernatant was collected and stored at −80°C until use. Colonic mucosal levels of SP, TNF- α and IL-6 were determined using ELISA kits according to the manufacturer’s protocol.

### Reverse transcriptase–polymerase chain reaction (RT–PCR)

Total RNA was extracted from colonic mucosa and pulmonary tissue using TRIzol reagent. RNA concentration was adjusted to 1 mg/ml with RNase-free distilled water. Reverse transcrip-tase–polymerase chain reaction (RT–PCR) was performed using an Access Quick RT–PCR System, which combined cDNA synthesis and PCR in a single reaction. Sub- sequnetly, 1 μl of total RNA was added to the 49 μl final reaction mixture solution. For single-step RT–PCR, reverse transcription (48°C for 50 min) was followed by initial denaturation at 95°C for 2 min. Cycling profiles used were: denaturing at 95°C for 30 s; annealing at 55°C for 1 min and extension at 72°C for 2 min. A total of 30 cycles was used, followed by a final extension step of 5 min at 73°C. The following oligonucleotide primers were designed: PAR-2, 5’-CACCAGTAAGGGAGAAGTCT-3’ (sense), 5’-GG GC AG CA CGTCGTGACAGGT-3’ (antisense); and β-actin, ’5-CGTGGGCCGCCCTAGGCACCA-3’ (sense) and 5’-TTGGCCTTAGGGTTCAGGGGG-3’ (antisense).

Suitable sizes of synthesised cDNA for PAR-2 and β-actin were 385 and 275 bp, respectively. PCR products in each cycle were electrophoresed on 2% agarose gel. PCR products of the predicted size were stained using ethidium bromide and visualized using an ultraviolet transilluminator. Quantification of each band was performed using Scion Image densitometry analysis software.

### Statistical analysis

Results are expressed as mean ± S.D. The effect of TXYF and LR was examined using one-way ANOVA and Dunnett’s test. Differences were considered significant when P < 0.05.

## Results

### Effects of TXYF on colon hypersensitivity

**Figure 1:**
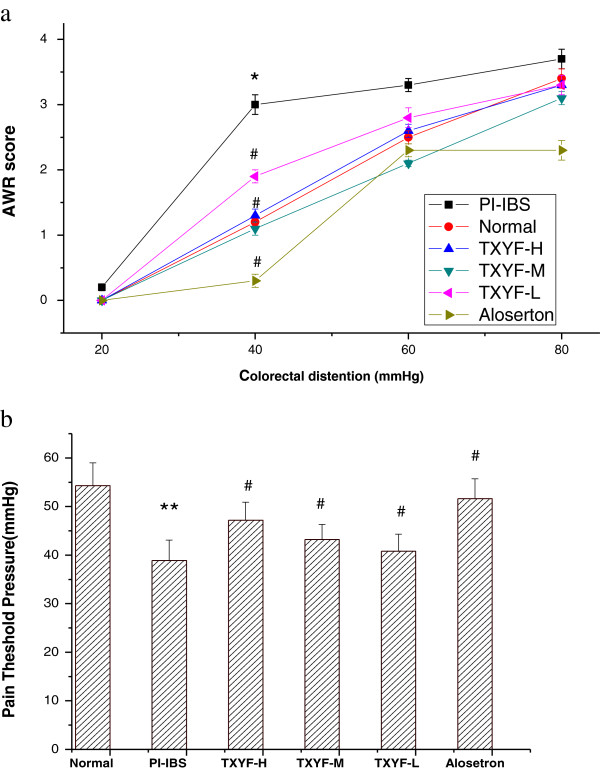
**Effects of TXYF on colon hypersensitivity. (a)** The effect of TXYF on AWR score in PI-IBS rats. Abdominal withdrawal reflex score (AWR score) measured in response to graded colorectal distension shows significant differences between the PI-IBS group and the normal group and between TXYF treatment group and the PI-IBS group at 40 mm Hg. Data are mean ± S.D. *P < 0.05 vs. Normal, ^#^P < 0.05 vs. PI-IBS. **(b)** The effect of TXYF on PTP in PI-IBS rats. Pain threshold pressure (PTP) measured showed significant decrease in the rats with visceral hypersensitivity comparing to the normal control group, and a significant increase in the TXYF at three dosage treatment group comparing to the PI-IBS group. Data are mean ± S.D. **P < 0.01 vs. Normal, ^#^P < 0.05 vs. PI-IBS.

As shown in Figure 1a, the AWR scores in response to graded CRD (20, 40, 60, and 80 mmHg) were significantly elevated in PI-IBS rats when compared to that of the normal rats (P < 0.05). TXYF significantly reduced AWR scores in PI-IBS rats.

As shown in Figure 1b, The PTP of PI-IBS rats in response to CRD was reduced when compared to that of the normal rats (P < 0.05). TXYF at three dosage significantly increased The PTP compared to that of PI-IBS rats (P < 0.05). These results suggest that TXYF has analgesic effects on PI-IBS rats in a dose-dependent manner.

### Effects of TXYF on frequency of stool

**Figure 2:**
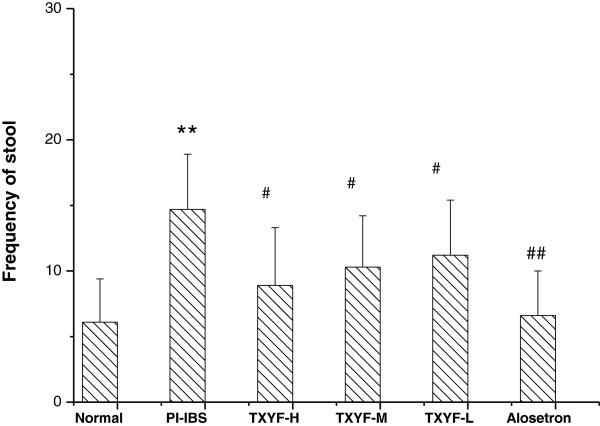
**The effect of TXYF on frequency of stool in PI**-**IBS rats.** Frequency of stool measured shows significant increase in the rats with the PI-IBS group comparing to the Normal group rats, and a significant decrease in TXYF treatment group comparing to the PI-IBS group. Data are mean ± S.D. **P < 0.01 vs. Normal; ^#^P < 0.05, ^##^P < 0.01 vs. PI-IBS.

As shown in Figure 2, the frequency of stool was significantly increased in PI-IBS rats when compared to that of the normal rats (P < 0.05), TXYF significantly reduced the frequency of stool in PI-IBS rats (P < 0.05).

### Effects of TXYF on faecal serine enzymatic activities

As shown in Table 2, the serine enzymatic activities were significantly increased in PI-IBS rats when compared to that of the normal rats (P < 0.05), TXYF significantly decreased the enzymatic activities in PI-IBS rats (P < 0.05).

**Table 2:** The effect of TXYF on faecal serine enzymatic activities

**Group**	**Dose (g/kg)**	**No. of rats**	**Serine activitie (mg/protein)**
Normal rats	-	10	772±236
PI-IBS rats	-	10	1956±411**
TXYF-H rats	4.8	10	1023±255^#^
TXYF-M rats	2.4	10	1254±289^#^
TXYF-L rats	1.2	10	1769±213
Alosetron rats	1.5×10^-3^	10	1235±256^#^

**p<0.01 vs Normal rats; ^#^p<0.05 vs PI-IBS rats.

### Effects of TXYF on SP, TNF- α and IL-6 expression in colonic mucosa

**Figure 3:**
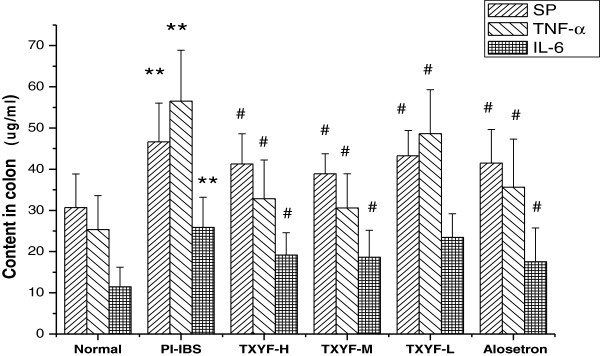
**The effect of TXYF on the contents of SP, TNF- α and IL-6 in colonic mucosa The contents of SP, TNF- α and IL-6 in colonic mucosa show significant differences between PI-IBS and Normal group and between TXYF treatment group and the PI-IBS group.** Data are mean ± S.D. **P < 0.01 vs. Normal, ^#^P < 0.05 vs. PI-IBS.

As shown in Figure 3, the contents of SP, TNF- α and IL-6 were significantly elevated in PI-IBS rats when compared to that of the normal rats (P < 0.05). TXYF treatment could significantly reduce the colonic mucosa contents of SP, TNF- α and IL-6 when compared to that of PI-IBS rats (P < 0.05).

### Effects of TXYF on PAR-2 expression in the colonic mucosa of PI-IBS rats

**Figure 4:**
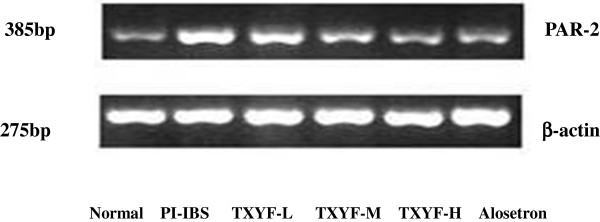
**The effect of TXYF on PAR**-**2 mRNA in colonic mucosa.**

As shown in Table 3 and Figure 4, relative quantification of PAR-2 mRNA in the colonic mucosa revealed that rats undergone PI-IBS had higher levels of PAR-2 mRNA (1.485 ± 0.537) than that of normal rats (0.634 ± 0.224) (P< 0.05). After TXYF treatment, relative expression quantification of PAR-2 mRNA in the colonic mucosa significantly decreased when compared to that of PI-IBS rats (P < 0.05). These results suggest TXYF has analgesic and anti-diarrhea effects on PI-IBS rats in a dose-dependent manner by down-regulating the expression of PAR-2 mRNA in the colonic mucosa.

**Table 3:** **The effect of TXYF on PAR**-**2 mRNA expression in the colonic mucosa of PI**-**IBS rats** (**n**=**10**)

**Group**	**Dose (g/kg)**	**Relative expression quantification of PAR**-**2mRNA**
Normal rats	-	0.634±0.224
PI-IBS rats	-	1.485±0.537^**^
TXYF-H rats	4.8	0.912±0.197^#^
TXYF-M rats	2.4	1.104±0.285^#^
TXYF- L rats	1.2	1.206±0.318
Alosetron rats	1.5×10^-3^	0.903±0.175^#^

**p<0.01 vs Normal rats; ^#^p<0.05 vs PI-IBS rats.

## Discussion

This study revealed that Chinese herbal formula TXYF and its componet herb LR could attenuate visceral hyperalgesia and reduce the frequency of stool in PI-IBS rats, which effect possibly mediated through down-regulating the PAR-2 mRNA expression in colonic mucosa.

IBS has a complex etiology and its pathogenesis is related to the altered gut sensory–m otor function, intestinal permeability, and infection, especially low-grade inflammation of the intestine [16]. Epidemiological studies show that bacterial gastroenteritis precedes the onset of the disorder in about 25% of patients with IBS [17,18]. Subtle immune activation has been reported in the colonic biopsies, and altered peripheral cytokine,such as IL-6 and TNF- α has been shown in PI- IBS patients [19].

Pathophysiological mechanisms of PI-IBS are linked with change in gut flora, altered small intestinal permeability, sensory-motor function and muscle hyper-contractility [20]. Recent studies suggest that low-grade inflammation plays important role in development of IBS following acute gastroenteritis [21,22], and neuroendocrine factors affect the motor function of gut [23]. Persistently increased number of inflammatory cells in IBS patents has been accompanied with the high expression of TNF- α and IL-6 mRNA even three months after an acute infective diarrhea [24]. Bood levels of IL-6 and the genetic polymorphism of TNF- α are significantly elevated in IBS patents [25,26]. TNF- α and IL-6 are regarded as the most important inflammatory cytokines in IBS. Moreover, the levels of IL-8、IL-10、IL-1 β and TGF-1β are significantly increased in IBS patents, especially IL-1 β expression is clearly elevated in post-infectious IBS (PI-IBS) [27].

PARs are coupled to G-proteins and cleaved by proteolysis, releasing a tethered ligand domain that binds and activates the receptor. PAR-2 has been reported to be activated by trypsin or mast cell tryptase. PAR-2 is distributed throughout the gastroin testinal tract, where it localized in epithelial cells, myocytes and enteric neurons [28]. Previous clinical data showed that PAR-2 activation is responsible for sensitization of sensory neurons, as well as visceral hypersensitivity in patiets with IBS. These results support the concept that PAR-2 is possibly an important receptor involved in the visceral nociceptive response and intestinal movement disorder. Reduced colonic microflora obtained by oral antibiotic treatment resulted in a lower serine protease activity and was associated with a decreased expression of PAR-2 on the colonic epithelial cells of mice [29]. In the present study, our results showed that the protease activity increased and the PAR-2 mRNA expression was up-regulated in PI-IBS rats. Moreover, TXYF could down-regulate the PAR-2 mRNA expression and inhibit the serine protease activtate.

Our data suggested that there were the obviosurluy increasment of SP, TNF- α and IL-6 contents in colonic mucosa of PI-IBS rats. These showed that PAR-2 was activated and then induced the realease of inflammation medium. TXYF could reduce the realease of SP, TNF- α and IL-6. In agreement with our present study, Gecse K found that increased faecals tryptase activity in IBS patent could induce visceral hypersensitivity in mice [28]. The mechanism for this phenomenon is that trypsin activates PAR-2 receptor increasing the excitability of dorsal root ganglion neurons and promoting SP and CGRP release. Tanaka Y found the infection factors of gastrointestinal can activate PAR-2 receptor promoting inflammation medium release, such as IL-6, IL-8 and TNF- α [30].

Previous research showed that TXYF can inhibit neonatal colon irritation-induced IBS by decreasing 5-HT in the serum and decreasing SP and CGRP in the plasma [31]. TXYF can treat IBS probably by affecting the secretion and release of gastrointestinal hormone (VIP) after the partial restriction stress binding stimulation in the IBS rat model [32]. TXYF has a marked inhibitory effect on the degranulation of peritonea1 mast cells in sensitized rats induced with C48/80, which indicates that TXYF exerts therapeutic effect by inhibiting enteric mast cell activation and thus decreasing histamine release, which in part explains the mechanism of TXYF in IBS [33].

In conclusion, our present research found that TXYF played an important role in attenuating behavioral hyperalgesia and anti-diarrheal effect. Down-regulating PAR-2 expression and reducing the level of cytokines in the colonic mucosa could be the possible molecular mechanism.

## Conclusions

Our study demonstrated that TXYF attenuated PI-IBS symptom by attenuating behavioral hyperalgesia and anti-diarrhea, the underlying mechanism was mediated by inhibiting PAR-2 receptor expression, reducing the levels of SP, TNF- α and IL-6 in colonic mucosa and decreasing faecal serine protease activity.

## Competing interests

The author’s declare that they have no competing interests.

## Authors’ contributions

XH designed the study and coordinated the experiments and draft of the manuscript. BH carried out the animal study. XZ participated in the experimental design and data analyses. WB conducted the extraction of RNA and RT-PCR. XH measured the contents of SP, TNF- α and IL-6 in colonic mucosal by ELISA. BH participated in the design and analysis of the experiment. XZ involved in discussion of experiment and draft of the manuscript. All authors read and approved the final manuscript.

## Pre-publication history

The pre-publication history for this paper can be accessed here:

http://www.biomedcentral.com/1472-6882/13/246/prepub
